# The Birth of Reproductive Health: A Difficult Delivery

**DOI:** 10.1371/journal.pmed.0010009

**Published:** 2004-09-07

**Authors:** Shereen El Feki

## Abstract

In 1994, the landmark "Cairo Conference" on population and development promised reproductive health for all. Ten years later, what has been achieved?

## Teaser

In 1994, the landmark Cairo Conference promised reproductive health and rights for all. Ten years later, what has been achieved?

About a decade ago, I went wandering around Cairo's City of the Dead. This might sound like a grim bit of tourism, but my connection to that vast necropolis runs deep—quite literally, as my family is buried there. After visiting their grave, I rambled through the city's dusty alleyways, past hundreds of years of history. Yet what I remember most about that day was not one of the many magnificent tombs, but a simple brick building with a sign, of all things, for a family planning clinic.

I was certainly surprised by my discovery; in retrospect, I should not have been. That part of Cairo is home to hundreds of thousands of people for whom looking after the dead is a way of life. Their fertility invigorates the funereal air: the caretaker of my family's tomb, for example, had a blooming family of his own living near the grave. Where better to offer family planning than in a place so poor that reproduction seemed more a matter of fate than choice?

## The Cairo Conference

That visit is a fitting metaphor for the field of reproductive health as a whole. Ten years ago, officials, experts, and activists from 179 countries also came to Cairo for the International Conference on Population and Development (ICPD). The conference produced a 20-year plan of action that focused on universal access to reproductive health services, including family planning and sexual health; reducing infant, child, and maternal mortality; better education, especially for girls; equality between men and women; and sustainable development.

The ICPD's key achievement was to reorient thinking on reproduction away from narrowly defined, government-dictated population control to a broader appreciation of reproductive and sexual well-being within health care systems, a view driven by individual choice and rights, not official priorities. “The Cairo Conference was a peak moment,” says Sally Ethelston, vice president for communications at Population Action International, one member of a consortium of non-governmental organisations launching a report card to mark the anniversary of the Cairo Conference in early September. “There were times when people were excited that they had accomplished something, and you could see it on their faces.”

Today, however, the mood is very different. While progress has been made on some of the plan's targets, effort has faltered on others. And the conference “camaraderie” that Ethelston describes has given way to conflict between faith and science, over abortion and condoms. Like signs of life in the City of the Dead, the Cairo Conference gave birth to great expectations, some of which have already expired.

## Baby Steps Towards Cairo's Goals

So, how far has the developing world come towards meeting the ICPD goals? There has certainly been progress on institutional reform in some countries, according to a recent survey of national policies by the United Nations Population Fund (UNFPA) [1]. For example, more than a third of the 151 countries questioned have introduced legislation on reproductive rights, and almost half have expanded their primary health care services to include family planning.[Fig pmed-0010009-g001]

**Figure pmed-0010009-g001:**
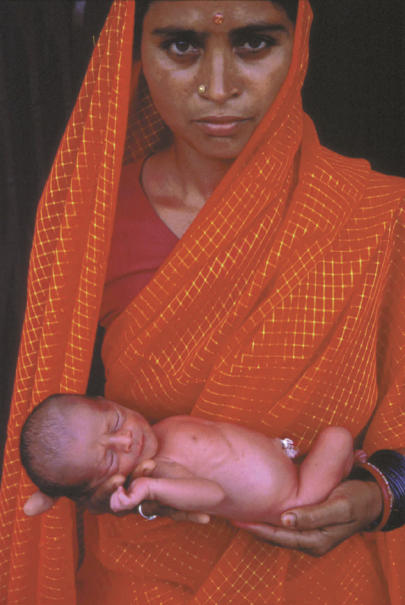
The birth of a baby on August 15, 2000, brought India's population to one billion (Photo: Raghu Rai, on behalf of the David and Lucile Packard Foundation)

But translating policy into action has been difficult. Overall, the picture is one of patchy success, according to Susheela Singh, director of research at The Alan Guttmacher Institute, a nongovernmental research organisation. Official statistics, as limited as they are for many aspects of reproductive and sexual health, show mixed results. On a positive note, global population growth has slowed to roughly 77 million people a year [2]. But while fertility rates have fallen in some developing countries, such as Mexico, they remain stubbornly high in others, such as Ethiopia [3]. Over the past decade, contraceptive use has grown, but so has demand, and there are now an estimated 201 million women in developing countries whose need for modern birth control goes unmet, resulting in 60 million unintended pregnancies a year [4]. Progress on legalising abortion has been slow, and an estimated 19 million abortions a year still occur under unsafe conditions [5]. Despite growing awareness of sexually transmitted disease, the annual number of sexually transmitted infections remains worryingly high at 340 million worldwide [6].

While infant mortality rates have improved somewhat, maternal mortality figures have barely budged. An estimated 529,000 women still die every year from complications of pregnancy and childbirth. The highest rates are in sub-Saharan Africa, where, on average, 920 women die for every 100,000 live births, compared with 24 deaths per 100,000 live births in Europe [7]. This is all the more distressing, says Vivien Tsu, senior programme officer at the Program for Appropriate Technology in Health, because these women's lives could be saved through straightforward measures and basic technologies, such as access to skilled midwives, simple drugs like magnesium sulphate for eclampsia and oxytocin for post-partum bleeding, cellular phones to call for help, and transportation to emergency obstetric centres.

## Obstacles to Reproductive Health

So why hasn't more been achieved? One problem is certainly money. The 1994 Cairo Conference estimated the cost of implementing programmes for family planning, maternal health, and prevention of sexually transmitted diseases, as well as data collection and analysis in developing countries, at $18.5 billion by 2005—or $24.3 billion in today's dollars. The goal was to mobilise one-third of the money from donor nations, and the rest from developing countries themselves [8].

Last year, global spending on reproductive health and services reached $14.7 billion, according to estimates from UNFPA, the Joint United Nations Programme on HIV/AIDS, and the Netherlands Interdisciplinary Demographic Institute [8]. Encouragingly, investment has increased since 2001, when the momentum of ICPD seemed to falter and international spending fell to $9 billion. But this is still wide of the mark. While developing countries have failed to meet their conference commitments, it is donor countries that are most remiss: rich country contributions reached an estimated $2.3 billion in 2003 [8], a far cry from the conference target of $6.1 billion (or $8.1 billion in today's dollars) by 2005.

Reproductive health is not alone in waiting for donors to give generously. For all the rhetoric at international summits, few rich countries have lived up to their lofty pledges of debt relief and of dedicating 0.7% of their gross domestic product to overseas development assistance. But as Steve Sinding, head of the International Planned Parenthood Federation (IPPF), points out, there are other reasons too for the shortfall. In the past donor interest was largely stimulated by fears of a population crisis. When the Cairo Conference reframed issues in terms of women's health and reproductive rights, rather than an impending population explosion, Sinding argues, the “demographic rationale” was lost, taking funding with it.

Moreover, there are other issues competing for international funding, most notably AIDS. At the time of the Cairo Conference, 20 million people were infected with HIV; today the number has grown to an estimated 38 million [9]. AIDS threatens to derail the Cairo Conference plan of action. Through maternal-to-child transmission, and wide-scale orphaning, HIV threatens to reverse small successes at reducing infant and child mortality. By killing off teachers and sapping household incomes, AIDS is sabotaging education. By killing off scarce medical workers and overwhelming fragile health care systems, the disease is compromising reproductive health services. Gender equity is undermined, as women and girls bear the brunt of the epidemic, as caregivers, breadwinners, or patients themselves.

Roughly half of the money spent on reproductive health last year went towards HIV/AIDS. And billions more is on the way, from the likes of the Global Fund to Fight AIDS, Tuberculosis, and Malaria and the United States President's Emergency Plan for AIDS Relief, which promises $15 billion over five years to HIV/AIDS programmes [10]. But much of this money is going into AIDS-specific programmes that do not address reproductive health more broadly. Even as the world is gearing up to scale up AIDS prevention and treatment to millions worldwide, few of the agencies involved come from the world of reproductive and sexual health.

This is a pity because it means that HIV/AIDS programmes are not making use of valuable infrastructure and expertise already on the ground in places where AIDS hits hardest. Given that 57% of HIV infections in sub-Saharan Africa are among women [9], and that, for many of them, family planning clinics are their sole contact with the formal health care system, it seems odd not to integrate such services into the wider battle against HIV. Such centres can offer not only HIV testing and counselling, as well as condoms (against the double whammy of unwanted pregnancy and HIV infection), but also a broad-based message of good sexual health that can help protect against HIV and other sexually transmitted diseases. Moreover, pre- and ante-natal care provide an opportunity to stop mother-to-child transmission of HIV in its tracks.[Fig pmed-0010009-g002]

**Figure pmed-0010009-g002:**
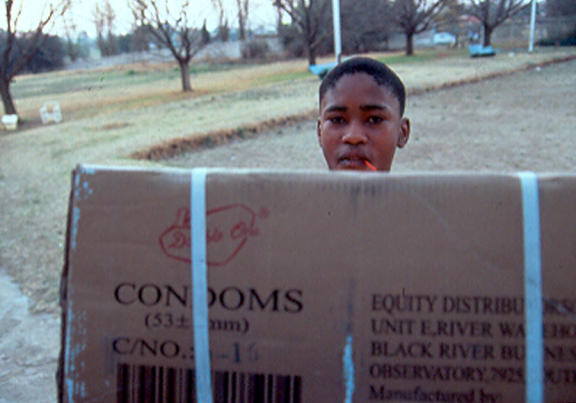
Condom distribution in Soweto, South Africa (Photo: Arjen van de Merwe, Population Concern)

Where once family planning was the darling of international donors, HIV is now the cause célèbre. “There's a lot of resentment about the spotlight moving on,” says Kevin O'Reilly, a former reproductive health specialist now at the department of HIV/AIDS at the World Health Organization. However, there are now attempts to bring the two together. Meetings earlier this year in Switzerland, New York, and Bangkok have led to calls to action to strengthen links between programmes addressing HIV/AIDS and sexual and reproductive health. While this should help in the battle against AIDS, the money which flows to AIDS should also benefit reproductive health.

## Ideological Battles

Arguably the most formidable obstacle to that union, and indeed further progress in improving reproductive health, is ideology. Since the Cairo Conference, a fierce battle has emerged between religious conservatives who eschew abortion and condoms in favour of abstinence and fidelity, and more liberal voices who argue for a full armamentarium to tackle these problems. The clash is loudest in the field of HIV/AIDS, where the President's Emergency Plan for AIDS Relief allocates a third of its funding for disease prevention to programmes focusing on abstinence and fidelity; public health experts argue that such an approach is ineffective at best, and dangerous at worst, without an equal emphasis on the availability of condoms for all.

But the clash resounds in the wider arena of reproductive health as well. Four years ago, the ICPD's central target—access to reproductive services for all by 2015—failed to make it into the Millennium Development Goals, largely because of political nervousness. But as Kofi Annan, United Nations secretary-general, has pointed out, progress on the other key targets, such as eradication of poverty and hunger, will not be achieved without a focus on women's rights, education, reproductive health, and family planning.

The fight between conservatives and liberals is clearest in the case of the US, which is the world's leading bilateral donor on reproductive health, spending $429 million this year [11]. However, this money comes with strings attached, says Françoise Girard, a reproductive rights lawyer in New York. Some of these are subtle. For example, Girard points to American pressure on several Asian and Latin American governments—during recent regional meetings to mark the anniversary of the Cairo Conference—not to re-affirm their commitment to the ICPD plan of action, with its emphasis on a full suite of reproductive rights and services.

Other strings are more obvious. In 2001, George W. Bush reinstated the Mexico City Policy, otherwise known as the “Global Gag Rule”, which denies US family planning assistance—including money and contraceptive supplies—to any non-American group unless it certifies that it neither performs nor endorses abortion. IPPF, Marie Stopes International, and their local affiliates have been hard hit by the Rule, scaling back services in Kenya, Ghana, and elsewhere that offered essential health care to thousands of women and children.

Then there is the Kemp-Kasten Amendment, a piece of US legislation which prohibits US assistance to any organisation as deemed by the President that “supports or participates in the management of a program of coercive abortion or involuntary sterilization.” At the behest of conservative supporters, President Bush has used the amendment to withhold $34 million in annual congressional appropriations to the UNFPA for the past three years. The UNFPA says that the $34 million could have been used to prevent 2 million unintended pregnancies, 800,000 induced abortions, 4,700 maternal deaths, and 77,000 infant and child deaths.

The White House accuses UNFPA of abetting coercive reproductive practices in China—a claim which the UNFPA strenuously denies. Moreover, a number of international delegations, including one from the US State Department in 2002, have investigated the UNFPA's activities in China and failed to find evidence to support such allegations.

Fortunately, other donors are stepping in to fill the breach: earlier this year, for example, the United Kingdom announced it would raise its contribution to the UNFPA to £80 million over the next four years, as well as increase its support to IPPF by a third. But even if the shortfall is made up, the ill will such clashes have engendered cannot be so easily salved.

## A Call for Strong Leadership

Getting it right on reproductive health cannot wait another decade. The largest generation of young people in history—a whopping 1.2 billion aged 10–19 years—is entering adulthood [1]. They are making their sexual debut at ever earlier ages, against a backdrop of rising sexually transmitted diseases and growing social conservatism, which makes clear information, frank discussion, and free choice on abortion, contraception, and sexual health extremely difficult. More than ever, reproductive health needs strong leaders in rich and poor countries alike to mobilise both money and political commitment. Reproduction is a sexy subject; it is time the world again paid it the attention it deserves.

**Useful Links**

**The Cairo Conference:** http://www.iisd.ca/cairo.html

**Population Action International:** www.popact.org

**UNFPA:** www.unfpa.org

**Program for Appropriate Technology in Health:** www.path.org

**The Alan Guttmacher Institute:** www.guttmacher.org

**The Joint United Nations Programme on AIDS:** www.unaids.org

**Netherlands Interdisciplinary Demographic Institute:** www.nidi.nl

**IPPF:** www.ippf.org

**Global Fund to Fight AIDS, Tuberculosis, and Malaria:** www.theglobalfund.org

**The World Health Organization HIV/AIDS Programme:** www.who.int/hiv/en

## Abbreviations

ICPD: International Conference on Population and Development
IPPF: International Planned Parenthood Federation
UNFPA: United Nations Population Fund
